# 4-Ethylguaiacol Modulates Neuroinflammation and Promotes Heme Oxygenase-1 Expression to Ameliorate Brain Injury in Ischemic Stroke

**DOI:** 10.3389/fimmu.2022.887000

**Published:** 2022-07-01

**Authors:** Wen-Tsan Weng, Ping-Chang Kuo, Barbara A. Scofield, Hallel C. Paraiso, Dennis A. Brown, I-Chen Yu, Jui-Hung Yen

**Affiliations:** ^1^ Department of Microbiology and Immunology, Indiana University School of Medicine, Fort Wayne, IN, United States; ^2^ Department of Anatomy, Cell Biology and Physiology, Indiana University School of Medicine, Fort Wayne, IN, United States; ^3^ Department of Pharmaceutical Sciences, Manchester University College of Pharmacy, Natural and Health Sciences, Fort Wayne, IN, United States

**Keywords:** 4-EG, HO-1, microglia, blood–brain barrier, ischemic stroke

## Abstract

Ischemic stroke is caused by a sudden reduction in cerebral blood flow that subsequently induces a complex cascade of pathophysiological responses, leading to brain inflammation and irreversible infarction. 4-ethylguaiacol (4-EG) is reported to suppress inflammatory immune responses. However, whether 4-EG exerts anti-inflammatory effects in ischemic stroke remains unexplored. We evaluated the therapeutic potential of 4-EG and examined the cellular and molecular mechanisms underlying the protective effects of 4-EG in ischemic stroke. The effect of 4-EG in ischemic stroke was determined by using a transient middle cerebral artery occlusion (MCAO) animal model followed by exploring the infarct size, neurological deficits, microglia activation, inflammatory cytokine production, blood–brain barrier (BBB) disruption, brain endothelial cell adhesion molecule expression, and microglial heme oxygenase-1 (HO-1) expression. *Nrf2^-/-^* and HO-1 inhibitor ZnPP-treated mice were also subjected to MCAO to evaluate the role of the Nrf2/HO-1 pathway in 4-EG-mediated protection in ischemic stroke. We found that 4-EG attenuated infarct size and neurological deficits, and lessened BBB disruption in ischemic stroke. Further investigation revealed that 4-EG suppressed microglial activation, peripheral inflammatory immune cell infiltration, and brain endothelial cell adhesion molecule upregulation in the ischemic brain. Finally, we identified that the protective effect of 4-EG in ischemic stroke was abolished in *Nrf2^-/–^* and ZnPP-treated MCAO mice. Our results identified that 4-EG confers protection against ischemic stroke and reveal that the protective effect of 4-EG in ischemic stroke is mediated through the induction of the Nrf2/HO1 pathway. Thus, our findings suggest that 4-EG could be developed as a novel therapeutic agent for the treatment of ischemic stroke.

## 1 Introduction

Stroke is ranked as the second leading cause of death worldwide with annual mortality more than 5 million. The burden of stroke not only lies on the high mortality but also high morbidity, resulting in up to 50% of survivors being chronically disabled (1). More than 80% of stroke cases belong to ischemic stroke, in which the occlusion of cerebral blood vessels initiates the acute phase of cerebral injury followed by neuronal excitotoxicity and oxidative damage. Following cerebral ischemia, the central nervous system (CNS) resident immune cells, microglia (MG), are rapidly activated in the injured brain. In addition, peripheral immune cells are recruited into the injured brain, leading to the secondary brain injury (2). In the CNS, MG maintain homeostasis; however, they can quickly respond to ischemic stress (3–5). Ischemic stress triggers MG activation and phagocytosis of damaged neurons in and around the infarct. Following reperfusion, macrophages play an important role in post-stroke inflammation in the ischemic brain (2). Infiltrating macrophages are activated and produce various inflammatory cytokines, including tumor necrosis factor alpha (TNFα) and Interleukin-1β (IL-1β) that promote neuronal cell death in the ischemic brain (5). In addition, there is a growing body of evidence demonstrating that blood–brain barrier (BBB) dysfunction is one of the major pathological causes following ischemic stroke (6). The function of BBB is known to protect the CNS and regulate peripheral leukocyte infiltration into the brain parenchyma through several molecules associated with brain endothelium (7). Among those regulatory molecules, tight junction proteins (TJPs), such as zonula occludens-1 (ZO-1) and occludin, and adhesion molecules, including intercellular adhesion molecule 1 (ICAM-1), vascular cell adhesion molecule 1 (VCAM-1), and E-selection, play important roles in regulating peripheral leukocyte recruitment and modulating the permeability of BBB following ischemic stroke (8).

4-ethylguaiacol (4-EG), a phenolic compound with the molecular formula C_9_H_12_O_2_, belongs to the class of organic compounds known as methoxyphenols (9, 10). 4-EG is produced along with 4-ethylphenol in wine and beer by the spoilage yeast Brettanomyces. In addition, 4-EG has been found in several different foods, such as green, yellow and orange bell peppers, corns, sesames, and coffee (10). Studies have demonstrated that phenolic compounds possess anti-inflammatory effects through inhibiting the expression of IL-1β, TNFα, IL-6, and cyclooxygenase-2 (9, 11). 4-EG has also been shown to exert anti-inflammatory effects through inhibiting nuclear factor kappa B (NFκB) and inflammasome activation as well as suppressing inflammatory cytokine production in THP-1 human monocytic cells (9, 11). Moreover, we have recently shown that 4-EG modulated neuroinflammation and inhibited Th1/Th17 differentiation to ameliorate disease severity in experimental autoimmune encephalomyelitis (EAE), the animal model of multiple sclerosis (12), further demonstrating the anti-inflammatory effect of 4-EG in the CNS diseases.

Currently, whether 4-EG possesses an anti-inflammatory effect on modulating acute neuroinflammation, such as ischemic stroke, remains unknown. Thus, we explored the potential protective effect of 4-EG in ischemic stroke and investigated the cellular and molecular mechanisms underlying the protective effect of 4-EG in ischemic stroke. We found that 4-EG attenuated brain injury, mitigated neurological deficits, and decreased mortality in mice subjected to ischemic stroke. Further investigations revealed that 4-EG inhibited MG activation and suppressed neuroinflammation in the ischemic brain. Moreover, 4-EG lessened BBB disruption, repressed the cell infiltration of the CNS, and suppressed brain endothelial adhesion molecule upregulation in ischemic stroke animals. Finally, mechanistic studies revealed that 4-EG induced HO-1 expression in MG, and the inhibition of the Nrf2/HO-1 pathway abolished the protective effect of 4-EG in ischemic stroke. Thus, our findings provide the first evidence that 4-EG could be developed as a novel therapeutic agent for the treatment of ischemic stroke.

## 2 Materials and Methods

### 2.1 Mice

All animal procedures were approved by the Purdue Animal Care and Use Committee and conducted in accordance with the National Institutes of Health Guidelines for the Care and Use of Laboratory Animals. C57BL/6 and *Nrf2^-/-^* mice were purchased from the Jackson Laboratory (Bar Harbor, ME, USA) and bred in the animal facility with controlled humidity, temperature, and 12 h:12 h light–dark cycle with free access to food and water. The genotyping was performed routinely to ensure the phenotypes of Nrf2 deficiency (**Supplementary Figure 1**).

### 2.2 Reagents

4-EG, triphenyltetrazolium chloride (TTC), Alexa Fluor 594 goat-anti rabbit IgG secondary antibody, Evans blue, Ficoll PM 400, and LPS (*Escherichia coli* O55:B5) were purchased from MilliporeSigma (St. Louis, MO, USA). Zinc protoporphyrin (ZnPP) was purchased from Alfa Aesar (Haverhill, MA, USA). 7-aminoactinomycin D (7-AAD), Alexa Fluor 488-conjugated anti-mouse CD45 (clone: 30-F11), APC-conjugated anti-mouse CD45 (clone: 30-F11), PE/Cy7-conjugated anti-mouse CD11b (clone: M1/70), PE-conjugated anti-mouse CD11b (clone: M1/70), PE/Cy7-conjugated anti-mouse CD86 (clone: GL-1), PE/Cy7-conjugated anti-mouse CD68 (clone: FA-11), Alexa Fluor 647-conjugated anti-mouse CD31 (clone: MEC13.3), PE/Cy7-conjugated anti-mouse ICAM-1 (clone: HA58), and PE/Cy7-conjugated anti-mouse VCAM-1 (clone: 429, MVCAM.A) antibodies for flow cytometer analysis were purchased from BioLegend (San Diego, CA, USA). PE-conjugated anti-mouse E-selectin (clone: 10E9.6) antibody for flow cytometry analysis was purchased from BD Biosciences (San Diego, CA, USA). Alexa Fluor 488 anti-mouse Iba1 (EPR16588) antibody for immunohistochemistry (IHC) was purchased from Abcam (Cambridge, MA, USA). Anti-mouse HO-1 (10701-1-AP) antibody for flow cytometer and IHC was purchased from Proteintech (Chicago, IL, USA).

### 2.3 Middle Cerebral Artery Occlusion Model

Male and female C57BL/6 and *Nrf2^-/-^* mice (8–12 weeks old) were used for cerebral ischemia experiments as previously described (13). The intraluminal suture occlusion model was carried out to induce transient ischemic stroke. Briefly, mice were anesthetized by isoflurane, and the body temperature was controlled at ~37 ± 0.5°C during the surgical procedure. Cerebral blood flow (CBF) was measured before, during, and after ischemia by laser Doppler flowmetry (Moor Instrument VMS-LDF2) at the parietal bone (2 mm posterior and 3 mm lateral from Bregma). The right common carotid artery (CCA) was clamped by a microvascular clamp, and the right external carotid artery (ECA) was exposed. A minimal incision was made in the ECA stump followed by the insertion of a silicon-coated 6.0 nylon monofilament (Doccol Corp, Sharon, MA, USA) through ECA to MCA. After 40 min (male) or 1 h (female) of occlusion, the intraluminal suture was removed to reestablish CBF. Mouse was then placed in the recovery cage with the temperature maintaining at 37°C for 1 h. Mice with a total reduction of CBF more than 75% were included and randomly assigned to different treatment groups. The sham surgery was conducted with the same procedures without the insertion of a suture. At 2 h post-reperfusion, 4-EG 100 mg/kg suspended in 100 µl phosphate-buffered saline (PBS) was i.v. administered to MCAO mice, and the same amount of PBS was i.v. administered to MCAO mice as vehicle controls. For the ZnPP treatment experiments, mice were i.p. administered vehicle (10% solutol) or ZnPP (30 mg/kg, dissolved in 10% solutol) overnight and 1 h prior to MCAO. At 2 h post-reperfusion, vehicle-treated MCAO mice were then subjected to vehicle or 4-EG 100 mg/kg treatment, and ZnPP-treated MCAO mice were subjected to 4-EG 100 mg/kg treatment. The investigators who conducted experiments were blinded to the animal groups.

### 2.4 Infarct Volume Measurement and Neurological Assessment

The cerebral infarct volume was measured to assess the severity of ischemic brain injury. Briefly, MCAO mice were anesthetized and transcardially perfused with PBS. The ischemic brain was then harvested and subjected to 2 mm coronal slicing with a rodent brain matrix followed by 1% TTC staining. After staining, the brain sections were scanned and the infarct volume was calculated by ImageJ as previously described (13). The neurological score was assessed at 48 h after injury using a six-point scale (0–5): score 0: normal; score 1: mild circling behavior with or without inconsistent rotation when picked up by the tail, <50% attempts to rotate to the contralateral side; score 2: mild consistent circling, >50% attempts to rotate to contralateral side; score 3: consistent strong and immediate circling, the mouse holds a rotation position for more than 1–2 sec with its nose almost reaching its tail; score 4: severe rotation progressing into barreling, the loss of walking, or righting reflex; and score 5: comatose or moribund.

### 2.5 Isolation of Mononuclear Cells From the Mouse Brain

Mononuclear cells were isolated from the mouse brain after ischemic stroke as previously described (14). MCAO mice were anesthetized and transcardially perfused with PBS. The brain was harvested and homogenized with 1X Hanks' balanced salt solution (HBSS) buffer followed by filtration through a 70-μm nylon cell strainer. After centrifugation, cells were resuspended in 30% Percoll underlayering with 70% Percoll. Following centrifugation, the mononuclear cells were then isolated from the interface between 30% and 70% Percoll. The isolated mononuclear cells were subjected to the surface staining of CD45 and CD11b to detect peripheral immune cell infiltrates or to the surface staining of CD45, CD11b, and CD86 in the presence of 7-AAD to assess MG activation. To detect CD68 expression, the mononuclear cells subjected to the surface staining of CD45 and CD11b in the presence of 7-AAD were fixed and permeabilized followed by CD68 staining. To measure HO-1 expression, the mononuclear cells subjected to the surface staining of CD45 and CD11b in the presence of 7-AAD were fixed and permeabilized and then incubated with the primary HO-1 antibody followed by the Alexa Fluor 546 secondary antibody. The stained cells were then analyzed by flow cytometer (BD FACSVerse).

### 2.6 Immunohistochemistry

Brain samples were sectioned and fixed with 4% paraformaldehyde in PBS at 4°C overnight. After 6% and 30% sucrose dehydration, brain sections were embedded in optimal cutting temperature compound and cut into 16 μm cryosections. Sections were then permeabilized in PBS containing 0.5% Triton X-100 for 30 min. Following blocking with goat serum (5% goat serum and 0.25% Triton X-100 in PBS) at room temperature (RT) for 1 h, sections were incubated with the primary HO-1 antibody at 4°C overnight. After washing, sections were then stained with the secondary antibody for 2 h followed by the Alexa Fluor 488 anti-mouse Iba1 antibody staining for 2 h at RT. After washing, samples were coverslipped with ProLong Gold anti-fade mountant containing DAPI. Immunofluorescence images were captured with a fluorescence microscope (×20x BX53, Olympus). The slides stained with only secondary antibodies served as negative controls. The number of Iba1^+^ cells per square millimeter was counted, and Iba1^+^ cells were determined based on the cells with the morphology of large cell body and short dendrites, representing the stage 3–5 of MG activation (15). To determine HO-1 fluorescence intensity, negative controls were used to set up the color threshold of HO-1 fluorescence signals, and the mean of HO-1 fluorescence signals (fluorescence intensity) detected on the brain slides prepared from vehicle- and 4-EG-treated MCAO mice was calculated by ImageJ.

### 2.7 Evans Blue Extravasation Assay

The BBB permeability was assessed based on the leakage of Evans blue as previously described (14). Briefly, mice were i.v. injected with 2% Evans blue solution (4 ml/kg) at 2.5 h after reperfusion. After 1 h of Evans blue circulation, animals were anesthetized and transcardially perfused with PBS to remove intravascular Evans blue. The brains were then harvested, sliced, and scanned. The hemispheres were then separated and homogenized in 50% trichloroacetic acid solution. After centrifugation, supernatants were harvested and diluted with 95% ethanol in a ratio of 1:3. The amount of extravascular Evans blue in the supernatant was then determined by measuring the fluorescence with excitation at 540/25 nm and emission at 645/40 nm (BioTek Synergy™ HT microplate reader).

### 2.8 Isolation of Microvasculature From the Mouse Brain

The microvasculature harvested from the ischemic brains was subjected to the analysis of adhesion molecule expression on brain endothelial cells as previously described (16). The brains harvested from MCAO mice were subjected to the removal of olfactory bulbs and cerebellum followed by homogenization by using an electrical drill (Milwaukee drill connected to the Staco Energy Products Autotransformer, 3PN1010B). The homogenized tissues were then mixed with an equal volume of 40% Ficoll solution to a final concentration of 20% Ficoll. Following centrifugation at 5,800x g, 4°C for 20 min, the pellet containing enriched microvessels was harvested and resuspended with 1X HBSS containing 0.5 mg/ml collagenase at 4°C for 20 min. After adding 10% fetal bovine serum medium to stop the reaction, the mixtures were subjected to filtration through a 70 μm nylon cell strainer followed by centrifugation. Cells were then collected and stained with anti-CD31 in the presence of anti-ICAM-1, anti-E-selectin, or anti-VCAM-1 antibodies followed by flow cytometry analysis.

### 2.9 Cell Culture

Primary MG were generated from P1–P2 neonatal mice as previously described (14). Briefly, cerebral cortical cells were harvested from P1–P2 neonatal mice and then plated in 75 cm^2^ culture flasks with a complete Dulbecco's modified eagle medium: nutrient mixture F-12 (DMEM/F12) medium. At day 4 and 8 after cell plating, the medium was removed and replenished with complete media containing 10 ng/ml of granulocyte-macrophage colony-stimulating factor (GM-CSF). At day 12, MG were harvested from supernatants after the flasks were shaken at 37°C for 30 min. The mouse microglial cell line BV2 cells were grown in 25 cm^2^ flasks. After the cells were grown to confluence, they were trypsinized and seeded on tissue culture plates for experiments. Primary macrophages were generated from bone marrow cells as previously described (17). Briefly, the bone marrow cells were cultured in a complete roswell park memorial institute (RPMI) 1640 medium containing 10 ng/ml of macrophage colony-stimulating factor (M-CSF). The cells were replenished with fresh media containing 10 ng/ml of M-CSF at day 3 and harvested at day 7 for experiments. The mouse brain endothelial cell line bEnd.3 cells were grown in 25 cm^2^ flasks. After the cells were grown to confluence, they were trypsinized and seeded onto tissue culture plates for experiments.

### 2.10 Statistical Analysis

All results are given as mean ± SEM. The sample size was determined to be adequate based on our previous studies and also prior literature using similar experimental paradigms. The normal distribution of the data in each group was confirmed by the Shapiro–Wilk normality test. Comparisons between two groups were done by an unpaired *t-*test, whereas comparisons among multiple groups were done by one-way ANOVA (one variable) or two-way ANOVA (two variables) followed by the Tukey *post-hoc* test. For samples that did not pass the normality test, comparisons among multiple groups were done by the Kruskal–Wallis test followed by Dunn’s multiple comparison test. The statistical significance was determined as *p<0.05*.

## 3 Results

### 3.1 4-EG Ameliorates Brain Injury and Increases Survival in Ischemic Stroke

To determine whether 4-EG offered protection against ischemic stroke, C57BL/6 mice were subjected to MCAO and then administered vehicle or different doses of 4-EG (50, 100, or 150 mg/kg). At 48 h post-injury, mice were sacrificed and the ischemic brains were harvested and subjected to TTC staining to determine the infarct volume. We observed that the dose of 4-EG 50 mg/kg did not confer protection against ischemic stroke (**Supplementary Figures 2A, B**). In contrast, the doses of 4-EG 100 and 150 mg/kg conferred comparable protection against ischemic stroke, as MCAO mice treated with 4-EG 100 and 150 mg/kg displayed a similar level of cerebral infarct (**Supplementary Figures 2A, B**). However, 4-EG 150 mg/kg exerted a toxic effect, resulting in increased mortality in MCAO mice (**Supplementary Figure 2C**). Thus, the dose of 4-EG 100 mg/kg was selected to carry out the rest of studies.

**Figure 1:**
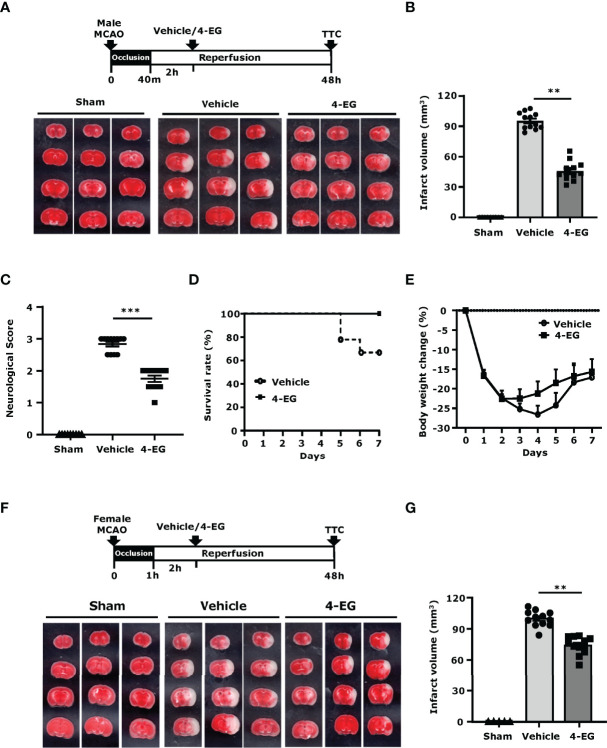
4-EG ameliorates brain injury and increases survival in ischemic stroke. **(A–E)** C57BL/6 male mice were subjected to sham or 40 min MCAO followed by vehicle or 4-EG (100 mg/kg) i.v. administration at 2 h post-reperfusion. **(A)** At 48 h post-injury, mice were sacrificed, and the ischemic brains were harvested and sliced (2 mm) followed by TTC staining. Three representative TTC-stained brain samples of sham, and vehicle- and 4-EG-treated MCAO mice are shown. **(B)** The infarct volumes and **(C)** neurological scores of sham (n=10), and vehicle- and 4-EG-treated MCAO mice (n=12/group) were evaluated. ^**^ *p*<*0.01*, ^***^ *p*<*0.001* by the Kruskal–Wallis test. **(D)** The survival rate and **(E)** body weight change of vehicle- and 4-EG-treated MCAO mice were evaluated up to day 7 post-injury (n=9/group). **(F, G)** C57BL/6 female mice were subjected to sham or 1 h MCAO followed by vehicle or 4-EG (100 mg/kg) i.v. administration at 2 h post-reperfusion. **(F)** Three representative TTC-stained brain samples of sham, and vehicle- and 4-EG-treated MCAO are shown, and **(G)** the infarct volumes of sham (n=5), and vehicle- and 4-EG- treated MCAO female mice (n=10/group) were assessed. ^**^ *p*<*0.01* by the Kruskal–Wallis test.

Upon the administration of MCAO mice with 4-EG 100 mg/kg, we observed that 4-EG not only attenuated cerebral infarct volumes (4-EG 45.7 ± 9.5 mm^3^ vs. vehicle 95.4 ± 8.1 mm^3^, **Figures 1A, B**) but also lowered neurological scores compared to vehicle-treated MCAO controls (**Figure 1C**). We further evaluated the long-term therapeutic effect of 4-EG on the survival of MCAO mice. Our results showed that 4-EG treatment increased long-term survival in MCAO mice in which vehicle-treated MCAO mice had a survival rate of 66.6%, whereas 4-EG-treated MCAO mice had a survival rate of 100% at day 7 post-injury (**Figure 1D**). We also compared the body weight changes between vehicle- and 4-EG-treated MCAO mice and observed that 4-EG-treated MCAO mice started to gain weight at day 3 post-injury, indicating that animals started to recover from ischemic brain injury. In contrast, vehicle-treated MCAO mice did not gain weight until day 5 post-injury and that could potentially contribute to decreased survival at day 7 post-injury (**Figure 1E**).

Finally, the effect of 4-EG in ischemic stroke was also evaluated in female C57BL/6 mice. Our results showed that 4-EG also conferred protection against ischemic brain injury in female MCAO mice, as 4-EG-treated female MCAO mice displayed a smaller infarct size compared to vehicle-treated female MCAO mice (4-EG 74.13 ± 8.6 mm^3^ vs. vehicle 99.9 ± 7.6 mm^3^, **Figures 1F, G**). Taken together, our results demonstrate that 4-EG attenuates ischemic brain injury, lessens neurological deficits, and increases survival in ischemic stroke animals, suggesting that 4-EG exerts a therapeutic potential for the treatment of ischemic stroke.

### 3.2 4-EG Suppresses MG Activation and Neuroinflammation in Ischemic Stroke

**Figure 2:**
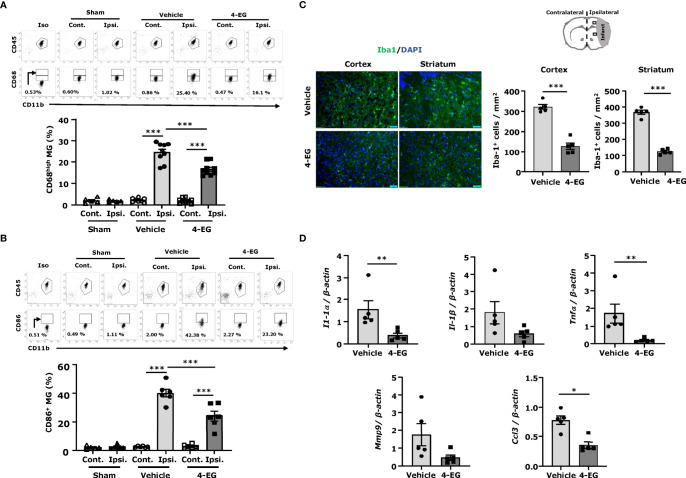
4-EG suppresses MG activation and neuroinflammation in ischemic stroke. C57BL/6 male mice were subjected to sham or 40 min MCAO followed by the i.v. administration of vehicle or 4-EG (100 mg/kg) at 2 h post-reperfusion. At 16–20 h post-injury, the ipsilateral and contralateral hemispheres of sham, and vehicle- and 4-EG-treated MCAO mice were harvested and subjected to mononuclear cell isolation. The isolated mononuclear cells were then stained with antibodies against CD45 and CD11b in the combination with CD68 or CD86 followed by flow cytometry analysis. MG were determined based on their surface intermediate expression of CD45 and positive expression of CD11b (CD45^int^CD11b^+^). Isotype controls (Iso) were used as a negative control to determine CD45^int^CD11b^+^ MG positive for CD68 or CD86 expression. **(A)** The gating of CD68 low (CD68^L^) was based on the basal expression of CD68 in MG in sham controls, and the expression level of CD68 higher than CD68^L^ was then determined as CD68 high (CD68^H^). The frequency of CD68^H^ MG in the contralateral and ipsilateral hemispheres of sham (n=5), and vehicle- and 4-EG-treated MCAO mice (n=9/group) was then measured and quantified. ^***^p<*0.001* by two-way AVONA. **(B)** The frequency of CD86 expression in the contralateral and ipsilateral hemispheres of sham (n=5), and vehicle- and 4-EG-treated MCAO mice (n=6/group) was also determined. ^***^p<*0.001* by two-way ANOVA. **(C)** At 20 h post-injury, the brain tissues of vehicle- and 4-EG-treated MCAO mice were harvested and subjected to IHC to assess Iba1 expression (n = 5/group). The representative images of Iba1^+^ cells in the ipsilateral cortex and striatum of vehicle- and 4-EG-treated MCAO mice are shown. Cell nuclei were stained by DAPI. Scale bar, 50 μm. The number of Iba1^+^ cells in the ipsilateral cortex and striatum was quantified. ^***^p < *0.001* by unpaired *t*-test. **(D)** The mRNA expression of IL-1α, IL-1β, TNFα, MMP9, and CCL3 in the ischemic brain of vehicle- and 4-EG-treated MCAO mice (n=5/group) was measured. ^*^p<*0.05,* ^**^ *p*<*0.01* by the Kruskal–Wallis test.

In response to ischemic cerebral injury, MG are rapidly activated and produce inflammatory cytokines and chemokines (18). Activated MG upregulate co-stimulatory molecules, such as CD80 and CD86 (19). Furthermore, CD68, a lysosomal protein, is also highly upregulated in activated MG (20). To explore the effect of 4-EG on MG activation in ischemic stroke, we measured the expression of maturation markers, CD68 and CD86, on MG. The ischemic brains were harvested from sham, and vehicle- and 4-EG-treated MCAO mice followed by mononuclear cell isolation, and the isolated cells were then subjected to the staining of CD45 and CD11b in the combination with CD68 or CD86 followed by flow cytometry analysis. We determined the MG population in the isolated mononuclear cells based on their intermediate expression of CD45 (CD45^int^) and positive expression of CD11b (CD11b^+^), and the gating strategy of flow cytometry analysis is presented in **Supplementary Figure 3A**. Our results showed that a low level of CD68 expression (CD68^L^) was detected in MG isolated from the ipsilateral hemisphere of sham controls as well as the contralateral hemisphere of vehicle- and 4-EG-treated MCAO mice, suggesting that MG express a basal level of CD68 under non-inflamed conditions (**Figure 2A**). Notably, ischemic stroke enhanced the microglial expression of CD68 (CD68^H^) in the ipsilateral hemisphere of the ischemic brain. However, 4-EG was capable of suppressing ischemia-enhanced microglial CD68 expression in the ipsilateral hemisphere (**Figure 2A**). Furthermore, we observed that ischemia stroke increased CD86^+^ MG in the ipsilateral hemisphere of vehicle-treated MCAO mice compared to that of sham controls (**Figure 2B**). Consistent with its effect on CD68 expression in MG, 4-EG-treated MCAO mice exhibited a decreased frequency of CD86^+^ MG compared to vehicle-treated MCAO mice (**Figure 2B**). Moreover, we subjected ischemic brain tissues to IHC to assess the level of Iba1^+^ cells in vehicle- and 4-EG-treated MCAO mice. MG, displaying an activated amoeboid morphology with a large soma size and short branching processes, were observed in the ischemic brain of vehicle-treated MCAO mice. In contrast, MG, exhibiting a resting ramified morphology with a small soma size and long branching processes, were found in the ischemic brain of 4-EG-treated MCAO mice (**Figure 2C** left). Further quantification of IHC results revealed that the number of Iba1^+^ cells in the ipsilateral cortex and striatum was significantly lower in 4-EG-treated MCAO mice compared to vehicle-treated MCAO controls (**Figure 2C** right). Finally, we measured the expression of inflammatory molecules in the ischemic brain of vehicle- and 4-EG-treated MCAO mice. We found that 4-EG treatment suppressed the expression of inflammatory molecules, including IL-1α, IL-1β, TNFα, MMP9, and CCL3, in the ischemic brain (**Figure 2D**). Taken altogether, these results demonstrate that 4-EG exerts anti-inflammatory effects on the suppression of MG activation and inhibition of inflammatory cytokine expression, leading to attenuated neuroinflammation in ischemic stroke.

### 3.3 4-EG Alleviates BBB Disruption and Represses Peripheral Immune Cell Infiltration of the CNS in Ischemic Stroke

**Figure 3:**
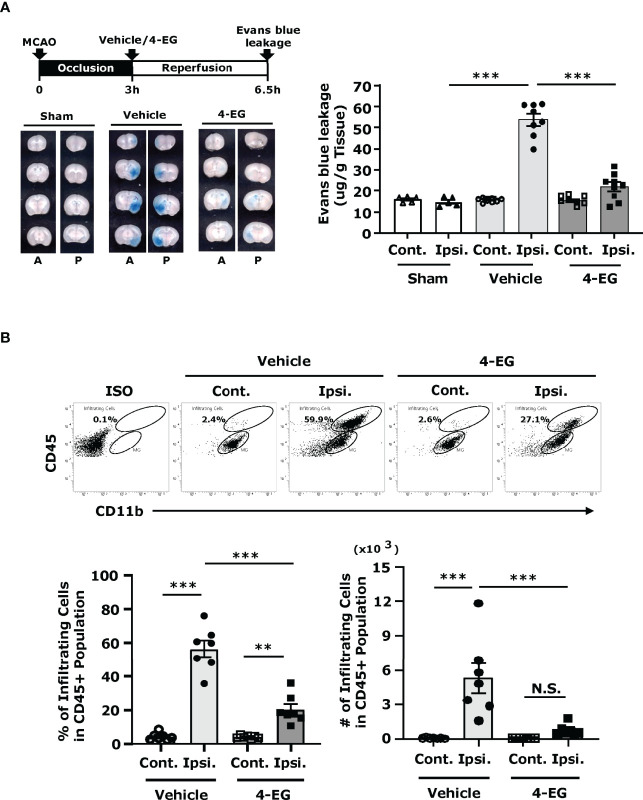
4-EG alleviates BBB disruption and represses peripheral immune cell infiltration of the CNS in ischemic stroke. **(A)** C57BL/6 male mice were subjected to sham or 3 h MCAO followed by vehicle or 4-EG administration. At 2.5 h post-reperfusion, mice were i.v.-administered Evans blue. 1 h after Evans blue injection, ischemic brains were harvested and sectioned. The representative brain images of sham, and vehicle- and 4-EG-treated MCAO mice are shown (A, anterior surface; P, posterior surface), and the Evans blue extravasation in the contralateral (Cont.) and ipsilateral (Ipsi.) hemispheres of sham (n=5), and vehicle- and 4-EG-treated MCAO mice (n=8/group) was determined. ***p<0.001 by two-way ANOVA. **(B)** C57BL/6 male mice subjected to 40 min MCAO were i.v. administered vehicle or 4-EG (100 mg/kg) at 2 h post-reperfusion. At 48 h post-injury, the ischemic brains harvested from vehicle- and 4-EG-treated MCAO mice were separated into the contralateral and ipsilateral hemispheres followed by mononuclear cell isolation (n=7/group). The isolated mononuclear cells were stained with antibodies against CD45 and CD11b and then analyzed by flow cytometry analysis. Isotype controls (Iso) were used as a negative control to determine CD45^int^CD11b^+^ MG and CD45^hi^CD11b^+^ infiltrating immune cells. The percentage and number of CD45^hi^CD11b^+^ infiltrating cells were then determined. ^**^p<*0.01,* ^***^ *p*<*0.001*, N.S., no significant differences by two-way ANOVA.

Following cerebral ischemia, BBB disruption plays an important role in causing neurological dysfunction in ischemic stroke (21). To determine whether 4-EG alleviated ischemia-induced BBB disruption, mice were subjected to MCAO with 3 h occlusion to induce a severe BBB disruption followed by the administration of vehicle or 4-EG, and the level of BBB disruption was determined by Evans blue leakage at 3.5 h post-reperfusion. We found that cerebral ischemia induced a severe BBB disruption, exhibiting a profound leakage of Evans blue in the ipsilateral hemisphere, whereas 4-EG treatment markedly mitigated ischemia-induced BBB disruption, displaying a significant reduction of Evans blue leakage in the ischemic brain (**Figure 3A**).

Reperfusion following cerebral ischemia recruits peripheral inflammatory immune cells into the ischemic brain that further exacerbates brain injury, leading to the secondary brain injury (22). To explore whether attenuated brain infarct and alleviated BBB disruption observed in 4-EG-treated MCAO mice were correlated with repressed peripheral immune cell infiltration of the CNS, the ischemic brains were harvested from vehicle- and 4-EG-treated MCAO mice and subjected to mononuclear cell isolation followed by flow cytometry analysis to assess the level of peripheral cell infiltrates in the ischemic brain. Our previous studies have demonstrated that the contralateral hemisphere of MCAO mice and sham controls displayed a similar pattern of cell infiltrates (23). Thus, the contralateral hemispheres could serve as a proper control as sham controls. We observed that the contralateral hemisphere of vehicle- and 4-EG-treated MCAO mice only had a very few cell infiltrates (**Figure 3B**). However, we found that the frequency and number of CD45^hi^CD11b^+^ monocytes/macrophages were significantly increased in the ipsilateral hemisphere compared to the contralateral hemisphere in vehicle-treated MCAO mice. In contrast, the frequency and number of CD45^hi^CD11b^+^ monocytes/macrophages were significantly decreased in the ipsilateral hemisphere of 4-EG-treated MCAO mice compared to that of vehicle-treated MCAO controls (**Figure 3B**). Taken altogether, our results demonstrate that 4-EG lessens ischemia-induced BBB disruption that may subsequently suppresses peripheral immune cell infiltration of the CNS, leading to alleviated reperfusion-induced secondary brain injury in ischemic stroke.

### 3.4 4-EG Inhibits Brain Endothelial Adhesion Molecule Expression in the Ischemic Brain

**Figure 4:**
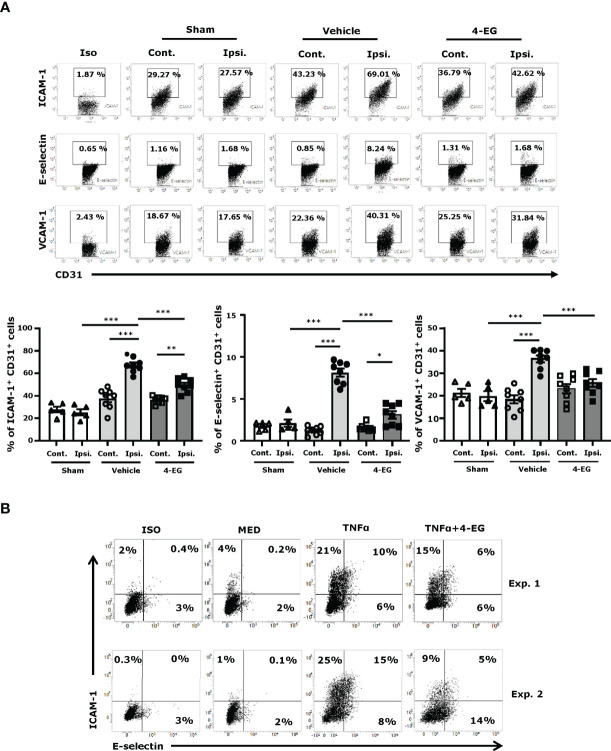
4-EG inhibits brain endothelial adhesion molecule expression in the ischemic brain. **(A)** C57BL/6 male mice were subjected to sham or 40 min MCAO followed by the i.v. administration of vehicle or 4-EG (100 mg/kg) at 2 h post-reperfusion. At 16–20 h post-injury, the contralateral and ipsilateral hemispheres of sham (n=5), and vehicle- and 4-EG- treated MCAO mice (n=8/group) were harvested and subjected to microvasculature isolation. The isolated cells were then stained with CD31 in the combination with ICAM-1, E-selectin, or VCAM-1 followed by flow cytometry analysis. Isotype controls (Iso) were used as a negative control to determine CD31^+^ brain endothelial cells positive for ICAM-1, E-selectin, or VCAM-1 expression. The frequency of ICAM-1^+^, E-selectin^+^, and VCAM-1^+^ CD31^+^ cells was determined. *p<0.05, ^**^p<*0.01,* ^***^ *p*<*0.001* by two-way ANOVA. **(B)** bEnd.3 cells were pretreated with 4-EG 200 µM for 1 h followed by TNFα 50 ng/ml stimulation for 4 h. Cells were then harvested and stained with ICAM-1 and E-selectin followed by flow cytometry analysis. Two representative flow cytometry results of five independent experiments are shown.

Studies have shown that the adhesion molecules, such as E‐selectin, P‐selectin, ICAM‐1, and VCAM-1, are upregulated on the surface of brain endothelial cells within hours after ischemic stroke that promotes the influx of inflammatory cells into the ischemic brain (24, 25). To explore whether 4-EG modulated brain endothelial adhesion molecule expression, the ischemic brains harvested from sham controls, and vehicle- and 4-EG-treated MCAO mice were subjected to microvasculature isolation followed by flow cytometry analysis to assess the level of adhesion molecule expression on the surface of brain endothelial cells. The brain endothelial cells were determined based on their positive expression of CD31, and the gating strategy of flow cytometry analysis is presented in **Supplementary Figure 3B**. Our results showed that ischemic stroke increased the frequency of ICAM-1^+^, E-selectin^+^, and VCAM-1^+^ CD31^+^ brain endothelial cells in the ipsilateral hemisphere of vehicle-treated MCAO mice compared to that of sham controls and the contralateral hemisphere of vehicle-treated MCAO mice (**Figure 4A**). Notably, the frequency of ICAM-1^+^, E-selectin^+^, and VCAM-1^+^ CD31^+^ brain endothelial cells was decreased in the ipsilateral hemisphere of 4-EG-treated MCAO mice compared to that of vehicle-treated MCAO mice **(** **Figure 4A**). The modulatory effect of 4-EG on adhesion molecule expression was further confirmed in the brain endothelial cell line, bEnd.3 cells, *in vitro*. We found that ICAM-1 and E-selectin were upregulated by TNFα stimulation in bEnd.3 cells. In contrast, 4-EG suppressed TNFα-upregulated ICAM-1 and E-selectin expression in bEnd.3 cells (**Figure 4B**). Taken altogether, our results demonstrate that 4-EG suppresses the upregulation of adhesion molecule expression in the brain endothelial cells of MCAO mice *in vivo* and in TNFα-activated bEnd.3 cells *in vitro*.

### 3.5 4-EG Promotes HO-1 Expression in MG *In Vivo* and *In Vitro*

**Figure 5:**
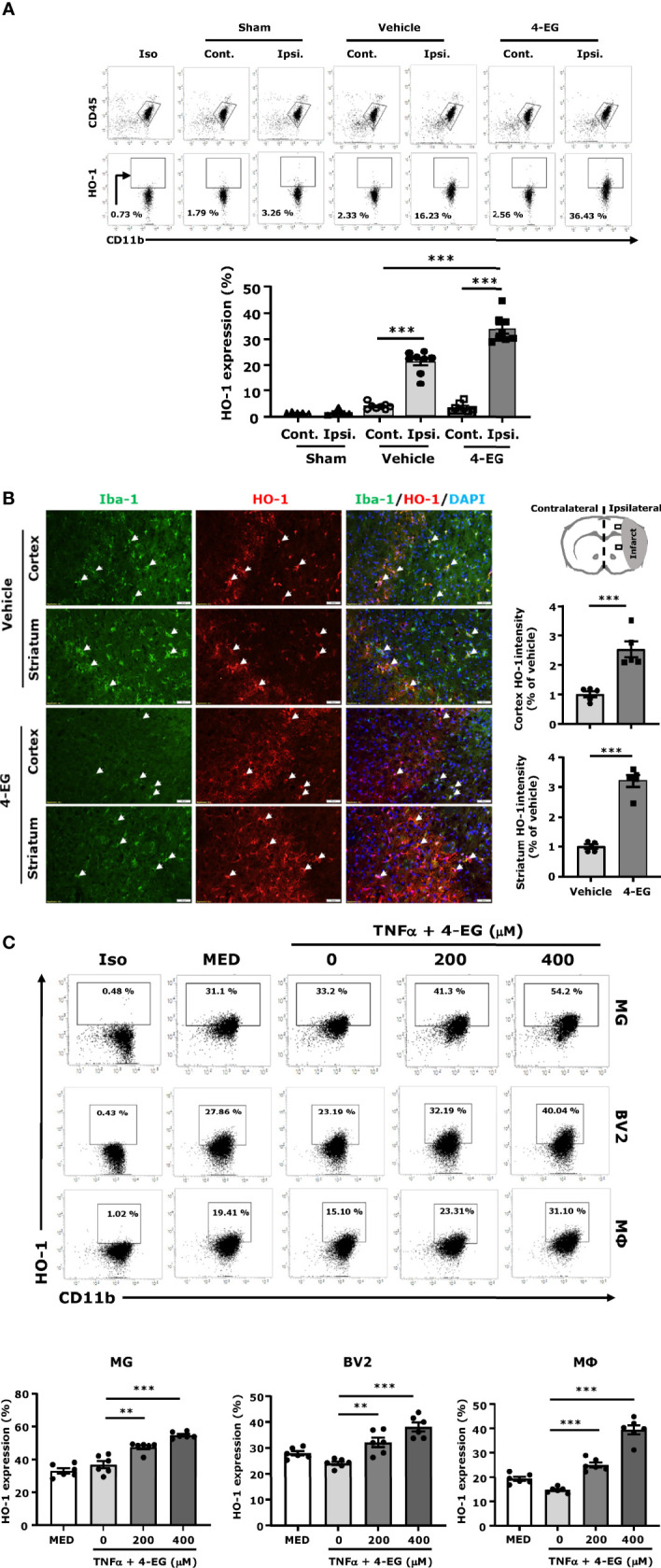
4-EG promotes HO-1 expression in MG *in vivo* and *in vitro*. **(A)** C57BL/6 male mice were subjected to sham or 40 min MCAO followed by vehicle or 4-EG (100 mg/kg) i.v. administration at 2 h post-reperfusion. At 16–20 h post-injury, the contralateral and ipsilateral hemispheres of sham (n=5), vehicle- and 4-EG-treated MCAO mice (n=8/group) were harvested followed by mononuclear cell isolation. The isolated mononuclear cells were subjected to the surface staining of CD45 and CD11b and then the intracellular staining of HO-1 followed by flow cytometry analysis. Isotype controls (Iso) were used as a negative control to determine CD45^int^CD11b^+^ MG positive for HO-1 expression. The frequency of HO-1 expression in CD45^int^CD11b^+^ MG was measured. ***p<*0.001* by two-way ANOVA. **(B)** At 20 h post-injury, the brain tissues of vehicle- and 4-EG-treated MCAO mice (n = 5/group) were subjected to IHC analysis to determine Iba1 and HO-1 expression. The representative images of Iba1 and HO-1 staining in the ipsilateral cortex and striatum of vehicle- and 4-EG-treated MCAO mice are shown. White arrows indicate the examples of Iba1/HO-1co-localization. The fluorescence intensity of HO-1 in the ipsilateral cortex and striatum was also quantified. Scale bar, 50 μm. ***p<*0.001* by unpaired *t-*test. **(C)** MG, BV2, and macrophages (MΦ) were pretreated with 4-EG 200 µM or 400 µM for 1 h followed by TNFα 100 ng/ml stimulation for 24 h (n=6/treatment group). Cells were then collected and subjected to the surface staining of CD11b and then the intracellular staining of HO-1 followed by flow cytometry analysis. Isotype controls (Iso) were used as a negative control to determine CD11b^+^ cells positive for HO-1 expression. **p<0.01, ***p<*0.001* by one-way ANOVA.

Previous studies have demonstrated that HO-1 overexpression significantly attenuates the infarct volume, and HO-1 deficiency results in exacerbated brain injury in ischemic stroke (26–28). Studies from other and our groups have shown that 4-EG induced HO-1 expression in THP-1 human monocytes and in the spinal cord of mice subjected to EAE, respectively (12, 29). Therefore, we explored whether 4-EG induced HO-1 expression in the ischemic brain to confer protection against ischemic stroke. As MG were reported to be the main producers of HO-1 in the CNS (30, 31), we therefore assessed whether MG displayed HO-1 expression following ischemic brain injury in vehicle- and 4-EG-treated MCAO mice. The ischemic brains were harvested from sham, and vehicle- and 4-EG-treated MCAO mice followed by mononuclear cell isolation, and the isolated cells were then subjected to the surface staining of CD45 and CD11b followed by the intracellular staining of HO-1. The expression of HO-1 in CD45^int^CD11b^+^ MG was determined by flow cytometry analysis, and the gating strategy of analysis is presented in **Supplementary Figure 3A**. We found that ischemic stroke induced HO-1 expression in MG in the ipsilateral but not contralateral hemisphere (**Figure 5A**), suggesting that cerebral ischemic insults induce oxidative stress that promotes HO-1 upregulation in the ischemic brain. Importantly, we found that 4-EG treatment further enhanced HO-1 expression in MG, as the frequency of HO-1-expressing MG was markedly increased in the ischemic brain of 4-EG-treated MCAO mice compared to that of vehicle-treated MCAO mice (**Figure 5A**). To further confirm the observed results, we subjected brain tissues to IHC to assess HO-1 expression. We observed HO-1 expression in the ipsilateral cortex and striatum of vehicle-treated MCAO mice. Consistently, 4-EG treatment further enhanced HO-1 expression in the ipsilateral cortex and striatum compared to vehicle treatment in MCAO mice (**Figure 5B**). Notably, HO-1 expression was co-localized with Iba1^+^ cells in the ischemic brain, although HO-1 expression was also observed in non-Iba1^+^ cells (**Figure 5B**). Finally, the effect of 4-EG on the induction of HO-1 was confirmed *in vitro* by using primary MG, BV2, and primary macrophages. Cells were activated with TNFα in the absence or presence of different doses of 4-EG followed by flow cytometry to determine HO-1 expression. The gating strategy of flow cytometry analysis is presented in **Supplementary Figure 4**. Our results showed that 4-EG dose-dependently upregulated HO-1 expression in these cells (**Figure 5C**). Altogether, our results demonstrate that 4-EG upregulates HO-1 expression in ischemia-activated MG *in vivo* and in TNFα-activated MG *in vitro*.

### 3.6 Inhibition of Nrf2/HO-1 Pathway Reverses the Protection Effect of 4-EG in Ischemic Stroke

**Figure 6:**
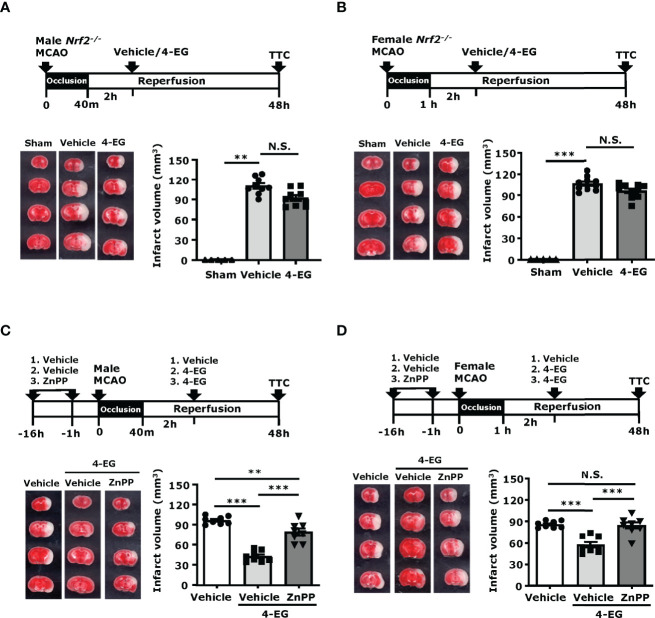
Inhibition of Nrf2/HO-1 pathway reverses the protection effect of 4-EG in ischemic stroke. **(A, B)** Male and female *Nrf2^−/−^* mice were subjected to sham or MCAO followed by vehicle or 4-EG (100 mg/kg) administration at 2 h post-reperfusion. At 48 h post-injury, mice were sacrificed, and the ischemic brains were harvested, sliced, and stained with TTC. **(A)**. The representative TTC-stained brain samples of sham, and vehicle- and 4-EG-treated male *Nrf2^−/−^* MCAO mice are shown (left panel), and the infarct volumes of sham (n=5), and vehicle- and 4-EG-treated male *Nrf2^−/−^* MCAO mice (n=9/group) were measured (right panel). ^**^ *p*<*0.01*, N.S., no significant differences by the Kruskal–Wallis test. **(B)** The representative TTC-stained brain samples of sham, and vehicle- and 4-EG-treated female *Nrf2^−/−^* MCAO mice are shown (left panel), and the infarct volumes of sham (n=5), and vehicle- and 4-EG-treated female *Nrf2^−/−^* MCAO mice (n=10/group) were measured (right panel). ^***^ *p*<*0.001*, N.S., no significant differences by the Kruskal–Wallis test. **(C)** Male and **(D)** female C57BL/6 mice were pretreated with either vehicle or ZnPP (30 mg/kg) overnight and 1 h prior to MCAO. At 2 h post-reperfusion, vehicle-treated MCAO mice were treated with vehicle or 4-EG, and ZnPP-treated MCAO mice were treated with 4-EG. At 48 h post-injury, mice were sacrificed, and ischemic brains were harvested and subjected to TTC staining. The representative TTC-stained brain samples are shown (left panel), and the infarct volumes of vehicle-, 4-EG-, and ZnPP+4-EG-treated MCAO (n=8/group) mice were measured (right panel). **p<0.01, ***p<0.001, N.S., no significant differences by one-way ANOVA.

To confirm whether the induction of the Nrf2/HO-1 pathway was required for the protective effect of 4-EG in ischemic stroke, male *Nrf2^-/-^* mice were subjected to MCAO followed by the administration of vehicle or 4-EG. 48 h post-injury, vehicle- and 4-EG-treated *Nrf2^-/-^* MCAO mice were sacrificed and the cerebral infarct was determined to assess the level of ischemic brain injury. Our results showed that the protective effect of 4-EG in ischemic stroke was reversed in *Nrf2^-/-^* MCAO mice, as vehicle- and 4-EG-treated *Nrf2^-/-^* MCAO mice exhibited a comparable level of cerebral infarct (vehicle 110.2 ± 12.5 mm^3^ vs. 4-EG 92.2 ± 12.4 mm^3^; **Figure 6A**). The reversed protective effect of 4-EG in *Nrf2^-/-^* MCAO mice was due to Nrf2 deficiency that subsequently led to attenuated HO-1 expression in the ischemic brain, as we observed 4-EG-induced HO-1 upregulation in MG was abolished in *Nrf2^-/-^* MCAO mice (**Supplementary Figure 5**). Furthermore, we determined the protective effect of 4-EG in female stroke mice with Nrf2 deficiency. Female *Nrf2^-/-^* mice were subjected to MCAO followed by vehicle or 4-EG treatment to determine the level of brain injury in ischemic stroke. Similarly, female vehicle- and 4-EG-treated *Nrf2^-/-^* MCAO mice displayed a comparable size of infarct (vehicle 106.2 ± 9.7 mm^3^ vs. 4-EG 96.2 ± 10.7 mm^3^; **Figure 6B**). Collectively, these results indicate that the activation of the Nrf2 pathway is essential for 4-EG-conferred protection against ischemic stroke.

To further confirm whether the induction of HO-1 was required for the protective effect of 4-EG in ischemic stroke, male and female mice were treated with vehicle or ZnPP, an HO-1 inhibitor, and then subjected to MCAO followed by 4-EG administration. The control MCAO mice were subjected to vehicle treatments only. At 48 h post-injury, MCAO mice were sacrificed and infarct volumes were determined. Our results showed that both male and female MCAO mice treated with ZnPP and 4-EG exhibited significant larger infarct volumes than MCAO mice treated with 4-EG only, but displayed slightly decreased or comparable infarct volumes compared to vehicle-treated male or female MCAO mice, respectively (**Figures 6C, D**). These results indicate that the inhibition of HO-1 by ZnPP reverses the protective effect of 4-EG in ischemic stroke. Taken altogether, our results demonstrate that the induction of the Nrf2/HO-1 pathway plays an essential role in 4-EG-conferred protection against ischemic stroke.

## 4 Discussion

In the present study, we demonstrate the efficacy of 4-EG treatment in ischemic stroke. Our *in vivo* results show that 4-EG treatment attenuates brain infarct, alleviates BBB disruption, improves neurological deficits, and increases survival in MCAO mice. At the cellular levels, 4-EG suppresses MG activation, leading to decreased CD86 and CD68 expression, and suppressed brain endothelial cell activation, resulting in reduced ICAM-1, E-selectin, and VCAM-1 expression in the ischemic brain. Mechanistically, 4-EG promotes HO-1 expression in MG to alleviate ischemic brain injury, as the protective effect of 4-EG in ischemic stroke is abolished in *Nrf2^-/-^* MCAO mice and MCAO mice treated with an HO-1 inhibitor. Thus, our results reveal that 4-EG exerts promising therapeutic effects on the attenuation of ischemic brain injury, suggesting that 4-EG could be developed as a novel therapeutic agent for the treatment of ischemic stroke.

Following ischemic stroke, neuroinflammation is a significant contributor to the pathological process of ischemic stroke (32–34). Inflammatogenic self-molecules derived from damaged tissue are generally called damage-associated molecular patterns (DAMPs), which are released from ischemic brain cells and stimulate DAMP receptors to induce the production of inflammatory cytokines and chemokines by MG that subsequently recruits peripheral immune cells infiltrating the injured brain (34). Thus, MG activation and peripheral immune cell infiltration contribute to the induction and aggravation of neuroinflammation in the ischemic brain, respectively (33, 35). In this study, we observed that 4-EG attenuated MG activation, as we found that the number of CD68- and CD86-expressing MG, and Iba1^+^ cells was largely reduced in 4-EG-treated MCAO mice compared to vehicle-treated MCAO controls. Furthermore, we observed a significant decrease of immune cell infiltrates in the ischemic brain of 4-EG-treated MCAO mice compared to that of vehicle-treated MCAO controls. Altogether, our results demonstrate that 4-EG suppresses MG activation and inhibits peripheral immune cell infiltration of the ischemic brain in MCAO mice, suggesting that the protective effects of 4-EG in ischemic stroke may be partly mediated through its modulatory effects on both the CNS and peripheral immune cells, leading to attenuated neuroinflammation.

Ischemic stroke–induced brain damage is a complex pathophysiological process including multichannel damages (36, 37). Studies have demonstrated that BBB dysfunction is a decisive event during the progression of stroke (38, 39). Because BBB integrity plays an important role in maintaining the microenvironment and homeostasis of the brain (40), protecting the BBB from disruption is a prospective strategy for the prevention and treatment of ischemic stroke. Adhesion molecules expressed on the surface of brain endothelial cells play a vital role in the recruitment of leukocytes, especially neutrophils, into the CNS after ischemic brain injury (21). Targeting these molecules to block immune cell recruitment and minimize secondary inflammatory responses in stroke has been a reliable strategy for ischemic stroke treatment (21, 41). Leukocyte migration involves the processes of rolling, adherence, and transendothelial migration, and these processes are needed for peripheral immune cells to access the ischemic brain through the BBB (21, 22). Cellular interactions between the endothelium and circulating leukocytes are mainly mediated by three groups of cell adhesion molecules, namely, selectins (such as E-selectin), the immunoglobulin superfamily (such as ICAM-1 and VCAM-1), and integrins (21, 41). The increased expression of E-selectin has been documented in the animal models of cerebral ischemia and has been shown to participate in neuroinflammation and brain injury after ischemic stroke (42). Furthermore, ICAM-1 has been shown to play a key role in psychiatric disorders, and it is a marker for inflammation (41). Moreover, studies have shown that the blockade of leukocyte adhesion by targeting the interactions among the various adhesion molecules prevents leukocytes from entering the ischemic tissue, resulting in reduced neuronal damage (12). In this study, we found that 4-EG treatment repressed the expression of ICAM-1, VCAM-1, and E-selectin in brain endothelial cells, and diminished Evans blue leakage in ischemic stroke. Taken altogether, our results suggest that 4-EG could potentially modulate cellular interactions between the endothelium and circulating leukocytes to improve BBB integrity after ischemic stroke.

Nrf2 plays a central role in cellular defense against oxidative stress (28, 43). Under the condition of redox imbalance, Nrf2 activation promotes the production of various detoxifying and antioxidant enzymes, including HO-1, NQO1, and Gclc, through binding to antioxidant response elements (AREs). It has been reported to HO-1 has the most AREs on its promoter, making it a promising therapeutic target against brain injury in ischemic stroke (28, 43). Notably, HO-1 knockout mice exhibited a larger infarct compared to their WT controls after ischemic stroke, and MCAO mice treated with adenoviral vector overexpressing HO-1, resulting in decreased infarct volumes and attenuated neurologic deficits (28). Since MG were reported to be the main producers of HO-1 in the CNS, we therefore conducted the assays of flow cytometry and IHC to determine the induction of HO-1 by 4-EG in MG following ischemic stroke. In the flow cytometry assay, MG were identified by their intermediate expression of CD45 and positive expression of CD11b, and their expression of CD45 and CD11b was not significantly altered following ischemic injury. We observed a low level of HO-1 expression in MG in both hemispheres of sham controls and the contralateral hemisphere of vehicle- and 4-EG treated MCAO mice, suggesting HO-1 is not induced under non-injured conditions in MG. In contrast, HO-1 was upregulated in MG in the ipsilateral hemisphere of vehicle-treated MCAO mice, and HO-1 expression was further upregulated in MG in the ipsilateral hemisphere of 4-EG-treated MCAO mice compared to that of vehicle-treated MCAO mice. These results demonstrate that ischemic insults induce HO-1 expression and 4-EG further upregulates HO-1 expression in MG in ischemic stroke. In the IHC assay, we found that Iba1 expression was downregulated in the ischemic brain of 4-EG-treated MCAO mice compared to that of vehicle-treated MCAO mice, suggesting that 4-EG suppresses MG activation. Notably, an increased level of HO-1 expression was observed in the ischemic brain of 4-EG-treated MCAO mice compared to that of vehicle-treated MCAO mice, and the co-localization of Iba1 and HO-1 immunoactivity was also detected in the ischemic cortex and striatum of vehicle- and 4-EG-treated MCAO mice. Collectively, these results demonstrate that 4-EG induces HO-1 upregulation in MG following ischemic stroke. Finally, we conducted *in vitro* studies to confirm our observation of the 4-EG-mediated induction of HO-1 expression *in vivo* by utilizing three different cell types, including primary MG, MG cell line BV2, and primary macrophages. Cells were activated with TNFα to mimic the inflammatory microenvironment in the ischemic brain, as previous studies have shown that TNFα was induced in the ischemic brain (44–46) and we also observed TNFα expression in the ischemic brain. Our results showed that 4-EG was capable of enhancing HO-1 expression in primary MG, BV2, and primary macrophages stimulated with TNFα. Altogether, our findings strongly demonstrate that 4-EG induces HO-1 expression in MG *in vitro* as well as *in vivo*.

Our observation of 4EG-induced HO-1 expression in the ischemic brain prompted us to investigate whether the induction of the Nrf2/HO-1 pathway was required for 4-EG-mediated protection against ischemic stroke. With the approach of inducing ischemic stroke in *Nrf2^-/-^* mice, we observed that the protective effect of 4-EG in ischemic stroke was reversed in *Nrf2^-/-^* MCAO mice. Importantly, we found that 4-EG-induced HO-1 upregulation in MG was abolished in *Nrf2^-/-^* MCAO mice. Furthermore, using the HO-1 inhibitor, ZnPP, we observed that the protective effect of 4-EG in ischemic stroke was also reversed in MCAO mice. Interesting, we noticed that the protective effect of 4-EG in ischemic stroke was not totally abolished in *Nrf2^-/-^* and ZnPP-treated MCAO mice, as 4-EG was still able to slightly attenuate brain infarct in *Nrf2^-/-^* and ZnPP-treated MCAO mice. These results suggest that the 4-EG-induced Nrf2/HO-1 pathway plays an essential role in alleviating brain injury induced by ischemic insults; however, 4-EG may induce additional protective mechanisms to offer protection against ischemic stroke. Indeed, 4-EG was previously reported to modulate NFκB and NLRP3 inflammasome activation (11), and the activation of NFκB and NLRP3 inflammasome was reported to induce brain injury in ischemic stroke (47, 48). Further studies would be required to investigate whether 4-EG directly inhibits NFκB and NLRP3 inflammasome activation to modulate brain injury in ischemic stroke.

There are limitations in our current study and that would be worthy of discussion. First, it is unknown whether 4-EG can cross the BBB, as there are no studies demonstrating their ability of crossing intact BBB. However, based on ChemDraw (PerkinElmer Informatics), 4-EG has a LogP value of 2.35 and it has been reported that drugs with LogP values above two are the most BBB penetrant (49). In addition, studies have shown that ischemic stroke induces BBB disruption (39), and in this study 4-EG was administered to MCAO mice at 2 h post-reperfusion that would facilitate their entering of the CNS through the disrupted BBB. Second, it is unknown what is the half-life of 4-EG *in vivo.* Although it is beyond the scope of the current study, future studies to investigate the pharmacokinetic properties of 4-EG would provide valuable data regarding its potential as a therapeutic agent for the treatment of ischemic stroke.

## 5 Conclusions

Our present study showed that 4-EG conferred protection against ischemic stroke. We observed that 4-EG suppressed MG activation, inhibited inflammatory molecule expression, and repressed the peripheral immune cell of infiltration of the CNS in ischemic stroke. Furthermore, we found that 4-EG suppressed brain endothelial cell adhesion molecule upregulation and alleviated BBB disruption in the ischemic brain. Finally, we revealed that 4-EG induced HO-1 expression in MG in the ischemic brain, and the inhibition of the Nrf2/HO-1 pathway reversed the protective effect of 4-EG in ischemic stroke. In summary, our results suggest that 4-EG, a natural compound, could be developed as a potential therapeutic agent for the treatment of ischemic stroke through its immunomodulatory and anti-inflammatory properties.

## Data Availability Statement

The original contributions presented in the study are included in the article/**Supplementary Material**. Further inquiries can be directed to the corresponding author.

## Ethics Statement

The animal study was reviewed and approved by Purdue Animal Care and Use Committee.

## Author Contributions

W-TW performed experiments, analyzed data, and wrote the manuscript. P-CK performed experiments and analyzed data. BS and HP performed experiments. DB and I-CY contributed to study discussion and manuscript editing. J-HY conceived the study, designed experiments, and wrote the manuscript. All authors read and approved the final manuscript.

## Funding

This work was supported by IU start-up fund and in part by NIH R01NS102449 to J-HY.

## Conflict of Interest

The authors declare that the research was conducted in the absence of any commercial or financial relationships that could be construed as a potential conflict of interest.

## Publisher’s Note

All claims expressed in this article are solely those of the authors and do not necessarily represent those of their affiliated organizations, or those of the publisher, the editors and the reviewers. Any product that may be evaluated in this article, or claim that may be made by its manufacturer, is not guaranteed or endorsed by the publisher.

## Supplementary Material

Supplementary Figure 1 Genotyping of *Nrf2^-/-^* mouse by PCR. Genomic DNA extracted from mouse tails of wildtype (WT) (n=2) and *Nrf2^-/-^* mice (n=8) was amplified by PCR with multi-primers for genotyping. The primers used are as follows: Forward primer: 5’-GCCTGAGAGCTGTAGGCCC-3’, WT reverse primer: 5’-GGAATGGAAAATAGCTCCTGCC-3’, mutant reverse primer: 5’-GACAGTATCGGCCTCAGGAA-3’. PCR products of 262 and 400 bp correspond to WT and *Nrf2^-/-^* alleles, respectively.

Supplementary Figure 2 The dose effect of 4-EG in ischemic stroke. C57BL/6 male mice were subjected to sham or 40min MCAO followed by vehicle or different doses of 4-EG (50, 100, or 150 mg/kg) i.v. administration at 2 h post-reperfusion (n=8/group). At 48 h post-injury, the ischemic brains were harvested and sliced (2 mm) followed by TTC staining. **(A)** Two representative TTC-stained brain samples of vehicle- and 4-EG-treated MCAO mice are shown, and **(B)** the infarct volumes of survived vehicle- and 4-EG-treated MCAO male were measured. ****p<0.001* by one-way ANOVA. **(C)** The survive rate of vehicle- and 4-EG-treated MCAO mice was also calculated.

Supplementary Figure 3 Gating strategy of flow cytometry analysis *in vivo.* **(A)** Mononuclear cells isolated from the brains of sham and MCAO mice were subjected to surface staining of CD11b and CD45 in the presence of 7-AAD followed by surface staining of CD86 or intracellular staining of CD68 or HO-1. 7-AAD negative live cells were then gated followed by singlet gating. CD45^int^CD11b^+^ cells were gated to identify MG, and isotype controls (Iso) were used as negative controls to determine CD45^int^CD11b^+^ MG positive for the expression of CD86, CD68, or HO-1. **(B)** Microvascular cells isolated from the brains of sham or MCAO mice were subjected to surface staining of CD31 in the presence of 7-AAD followed by surface staining of ICAM-1, E-selectin, or VCAM-1. 7-AAD negative live cells were gated followed by singlet gating. CD31^+^ cells were then gated to identify brain endothelial cells, and isotype controls (Iso) were used as a negative control to determine CD31^+^ cells positive for the surface expression of ICAM-1, E-selectin, or VCAM-1.

Supplementary Figure 4 Gating strategy of flow cytometry analysis *in vitro.*MG, BV2, and macrophages (MΦ) were subjected to surface staining of CD11b in the presence of 7-AAD followed by intracellular staining of HO-1. 7-AAD negative live cells were gated followed by singlet gating. Isotype controls (Iso) were used as negative controls to determine CD11b^+^ cells positive for the expression of HO-1.

Supplementary Figure 5 4-EG-induced HO-1 expression in MG is abolished in *Nrf2^-/-^* MCAO mice. *Nrf2^-/-^* male mice were subjected to 40 min MCAO followed by vehicle or 4-EG (100 mg/kg) i.v. administration at 2 h post-reperfusion (n=8/group). At 16-20 h post-injury, the contralateral and ipsilateral hemispheres of vehicle- and 4-EG-treated *Nrf2^-/-^* MCAO mice were harvested followed by mononuclear cell isolation. The isolated mononuclear cells were subjected to surface staining of CD45 and CD11b and then intracellular staining of HO-1 followed by flow cytometry analysis. Isotype controls (Iso) were used as a negative control to determine CD45^int^CD11b^+^ MG positive for HO-1 expression. The frequency of HO-1 expression in CD45^int^CD11b^+^ MG was measured. ^**^ *p*<*0.01;* ^***^ *p*<*0.001*, N.S; no significant differences by one-way ANOVA.

## Abbreviations

CNS, central nervous system; MG, microglia; 4-EG, 4-ethylguaiacol; BBB, blood–brain barrier; PBS, phosphate-buffered saline; 7-AAD, 7-aminoactinomycin D; HO-1, heme oxygenase-1; CBF, cerebral blood flow; CCA, common carotid artery; ECA, external carotid artery; IHC, immunohistochemistry; MCAO, middle cerebral artery occlusion; RT, room temperature; TJP, tight junction proteins; ZO-1, zonula occludens-1; TTC, triphenyltetrazolium chloride; ZnPP, zinc protoporphyrin.
