# Supplementary figures and images for: 4-Ethylguaiacol Modulates Neuroinflammation and Promotes Heme Oxygenase-1 Expression to Ameliorate Brain Injury in Ischemic Stroke
Source: Front Immunol. 2022 Jul 1;13:887000. doi: 10.3389/fimmu.2022.887000 (PMC9289724; doi:10.3389/fimmu.2022.887000)

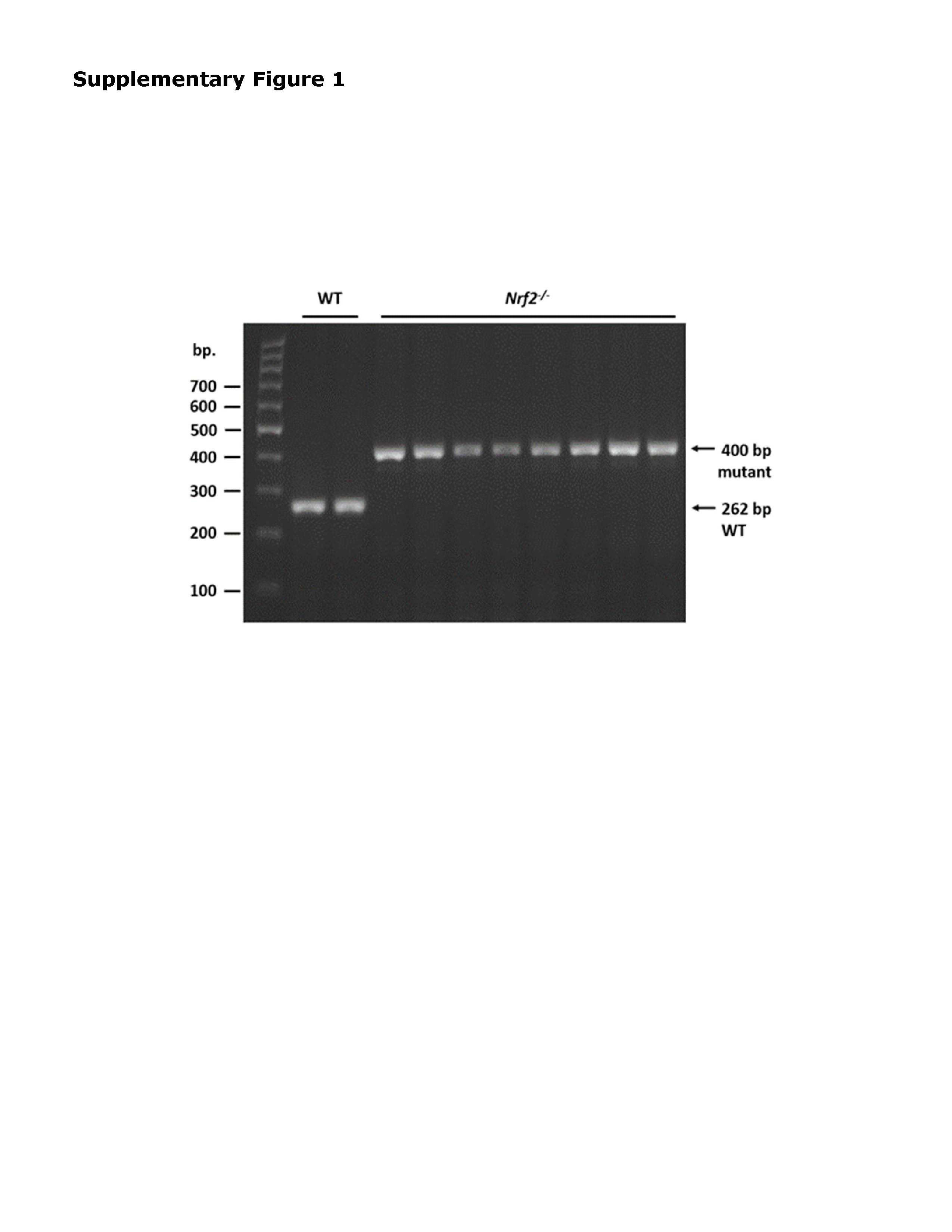

Supplement: Supplementary Figure 1 — Genotyping of Nrf2-/- mouse by PCR. Genomic DNA extracted from mouse tails of wildtype (WT) (n=2) and Nrf2-/- mice (n=8) was amplified by PCR with multi-primers for genotyping. The primers used are as follows: Forward primer: 5’-GCCTGAGAGCTGTAGGCCC-3’, WT reverse primer: 5’-GGAATGGAAAATAGCTCCTGCC-3’, mutant reverse primer: 5’-GACAGTATCGGCCTCAGGAA-3’. PCR products of 262 and 400 bp correspond to WT and Nrf2-/- alleles, respectively. [file Image_1.tif]

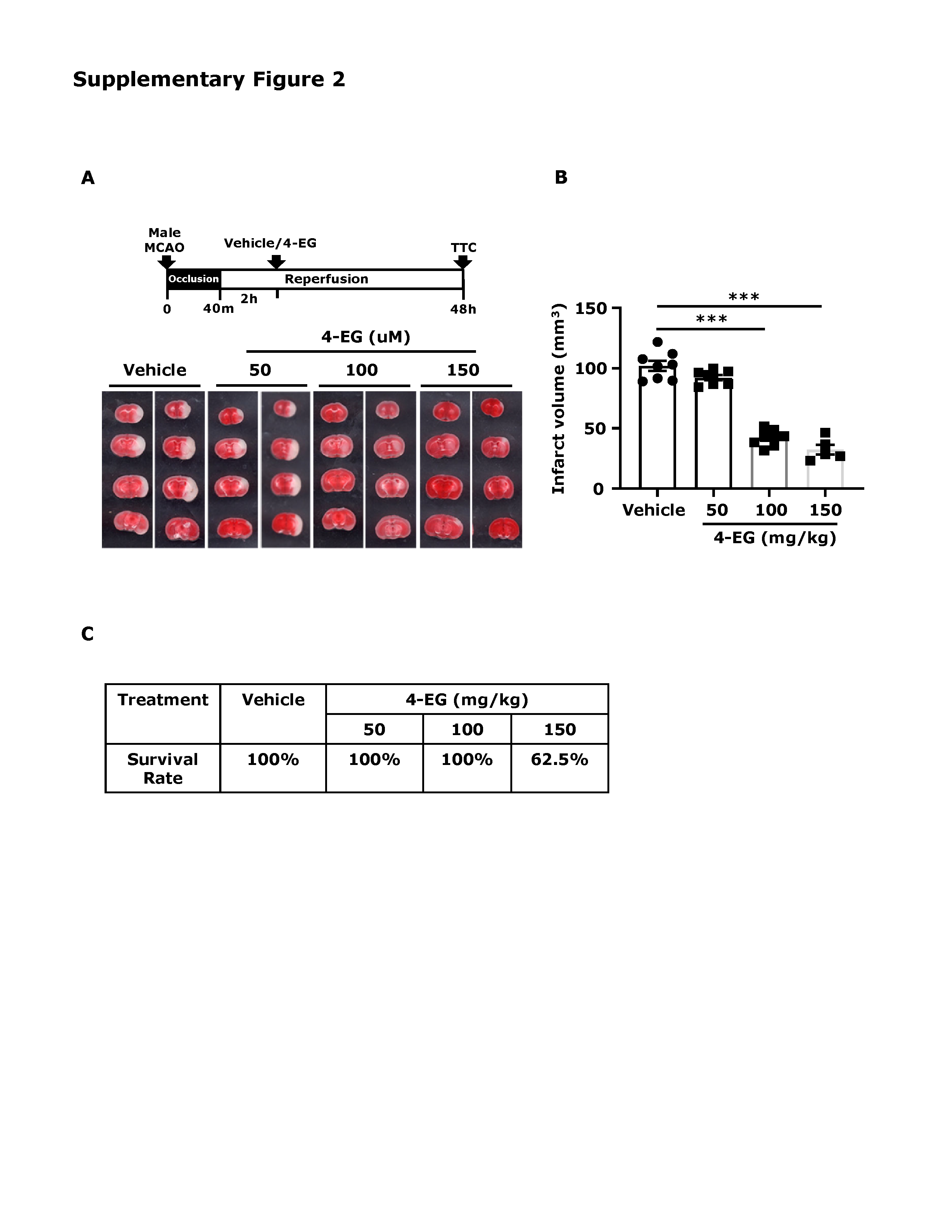

Supplement: Supplementary Figure 2 — The dose effect of 4-EG in ischemic stroke. C57BL/6 male mice were subjected to sham or 40min MCAO followed by vehicle or different doses of 4-EG (50, 100, or 150 mg/kg) i.v. administration at 2 h post-reperfusion (n=8/group). At 48 h post-injury, the ischemic brains were harvested and sliced (2 mm) followed by TTC staining. (A) Two representative TTC-stained brain samples of vehicle- and 4-EG-treated MCAO mice are shown, and (B) the infarct volumes of survived vehicle- and 4-EG-treated MCAO male were measured. ***p<0.001 by one-way ANOVA. (C) The survive rate of vehicle- and 4-EG-treated MCAO mice was also calculated. [file Image_2.tif]

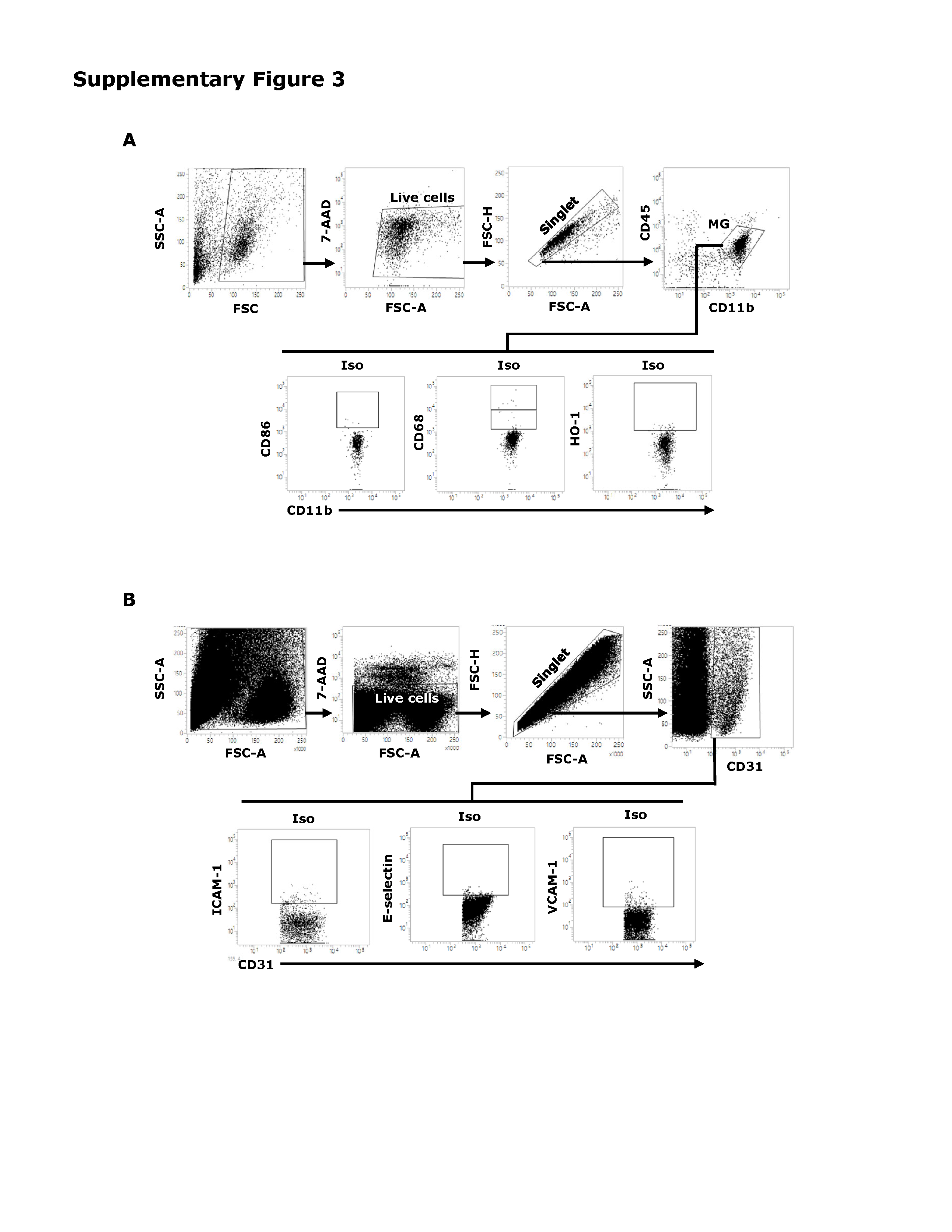

Supplement: Supplementary Figure 3 — Gating strategy of flow cytometry analysis in vivo. (A) Mononuclear cells isolated from the brains of sham and MCAO mice were subjected to surface staining of CD11b and CD45 in the presence of 7-AAD followed by surface staining of CD86 or intracellular staining of CD68 or HO-1. 7-AAD negative live cells were then gated followed by singlet gating. CD45intCD11b+ cells were gated to identify MG, and isotype controls (Iso) were used as negative controls to determine CD45intCD11b+ MG positive for the expression of CD86, CD68, or HO-1. (B) Microvascular cells isolated from the brains of sham or MCAO mice were subjected to surface staining of CD31 in the presence of 7-AAD followed by surface staining of ICAM-1, E-selectin, or VCAM-1. 7-AAD negative live cells were gated followed by singlet gating. CD31+ cells were then gated to identify brain endothelial cells, and isotype controls (Iso) were used as a negative control to determine CD31+ cells positive for the surface expression of ICAM-1, E-selectin, or VCAM-1. [file Image_3.tif]

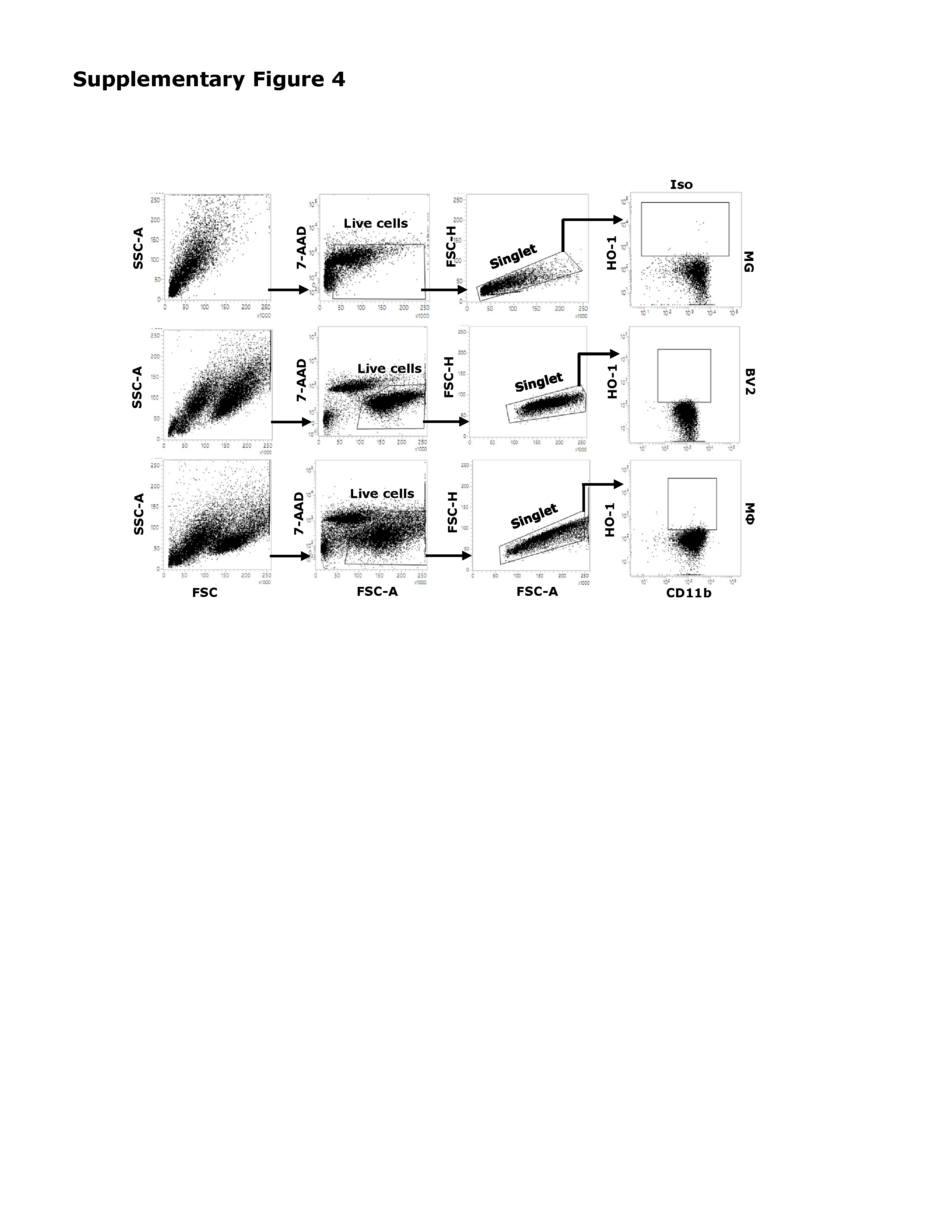

Supplement: Supplementary Figure 4 — Gating strategy of flow cytometry analysis in vitro.MG, BV2, and macrophages (MΦ) were subjected to surface staining of CD11b in the presence of 7-AAD followed by intracellular staining of HO-1. 7-AAD negative live cells were gated followed by singlet gating. Isotype controls (Iso) were used as negative controls to determine CD11b+ cells positive for the expression of HO-1. [file Image_4.tif]

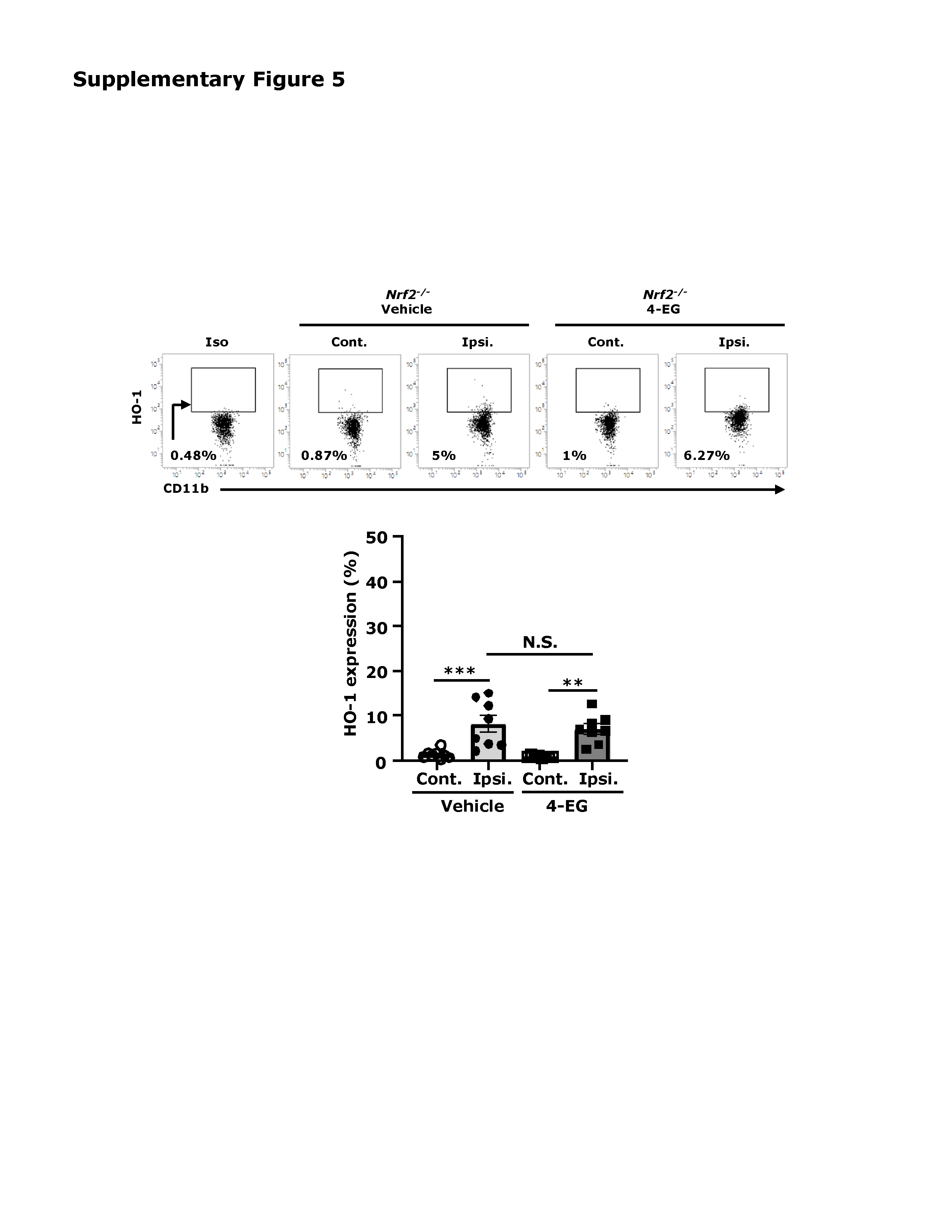

Supplement: Supplementary Figure 5 — 4-EG-induced HO-1 expression in MG is abolished in Nrf2-/- MCAO mice. Nrf2-/- male mice were subjected to 40 min MCAO followed by vehicle or 4-EG (100 mg/kg) i.v. administration at 2 h post-reperfusion (n=8/group). At 16-20 h post-injury, the contralateral and ipsilateral hemispheres of vehicle- and 4-EG-treated Nrf2-/- MCAO mice were harvested followed by mononuclear cell isolation. The isolated mononuclear cells were subjected to surface staining of CD45 and CD11b and then intracellular staining of HO-1 followed by flow cytometry analysis. Isotype controls (Iso) were used as a negative control to determine CD45intCD11b+ MG positive for HO-1 expression. The frequency of HO-1 expression in CD45intCD11b+ MG was measured. ** p<0.01; *** p<0.001, N.S; no significant differences by one-way ANOVA. [file Image_5.tif]
